# Source-dependent perlecan heparan sulfate chains in dECM modulate stem cell fate and multilineage differentiation

**DOI:** 10.3389/fbioe.2026.1930107

**Published:** 2026-10-21

**Authors:** Zhixin Wei, Qingqing Yu, Asif Shahriar, Xinzhou Xie, Song Chen, Ming Pei

**Affiliations:** 1 Stem Cell and Tissue Engineering Laboratory, Department of Orthopaedics, West Virginia University, Morgantown, WV, United States; 2 Southwest Jiaotong University, Chengdu, Sichuan, China; 3 Department of Spine Surgery, Jining No.1 People’s Hospital Affiliated to Shandong First Medical University & Shandong Academy of Medical Sciences, Jining, China; 4 Department of Orthopaedics, The General Hospital of Western Theater Command, Chengdu, Sichuan, China; 5 Tissue Stress Injury and Functional Repair Key Laboratory of Sichuan Province, The General Hospital of Western Theater Command, Chengdu, Sichuan, China; 6 Division of Exercise Physiology, West Virginia University, Morgantown, WV, United States; 7 WVU Cancer Institute, Robert C. Byrd Health Sciences Center, West Virginia University, Morgantown, WV, United States

**Keywords:** decellularized extracellular matrix, heparan sulfate, heparinase III, infrapatellar fat pad-derived stem cells, mesenchymal differentiation, perlecan

## Abstract

**Introduction:**

Perlecan, a heparan sulfate (HS) proteoglycan enriched in cell-derived decellularized extracellular matrix (dECM), carries N-terminal HS chains that act as co-receptor reservoirs for FGF-2, but whether this bioactivity is source-dependent is unknown.

**Methods:**

Passage-6 infrapatellar fat pad-derived stem cells (IPFSCs) were expanded on dECM from unmodified (dE) or SV40-immortalized (s-dE) IPFSCs, with or without heparinase III (HPA) to ablate HS chains, yielding five groups: PL (plastic), dE, dE(H), s-dE, and s-dE(H). Stemness, surface markers, and trilineage differentiation were assessed by RT-qPCR, histology, immunohistochemistry, and colorimetric assays over 21-day induction periods.

**Results:**

HPA selectively cleaved HS chains from matrix-resident perlecan without disrupting the core protein. Both dECM substrates suppressed adipogenic potential. HPA markedly enhanced adipogenesis in both groups. Chondrogenic and osteogenic responses were strikingly source-dependent: dE(H) preconditioning impaired chondrogenic differentiation, while s-dE(H) preconditioning conferred superior chondrogenic output—including the most prominent sGAG deposition and the largest pellet size among the groups examined—and exclusively promoted osteogenic matrix mineralization.

**Discussion:**

These findings indicate that perlecan-anchored HS chains are context-dependent regulators of stem cell fate, whose effects are modulated by dECM cellular origin, likely via structural differences in HS chains that differentially modulate FGF and BMP signaling. Targeted manipulation of matrix HS architecture offers a tunable strategy for dECM-based preconditioning in musculoskeletal regenerative medicine.

## 1 Introduction

Articular cartilage has a limited intrinsic capacity for self-repair, making cell-based therapies an attractive strategy for regenerative medicine. Mesenchymal stem cells (MSCs) are promising candidates owing to their self-renewal capacity and multilineage differentiation potential, including robust chondrogenesis (Toh et al., 2014). However, the extensive *in vitro* expansion required to generate therapeutic cell numbers progressively erodes MSC differentiation competence (Li and Pei, 2012), a limitation that must be overcome before MSC-based therapies can achieve clinical impact. Strategies that preserve or restore chondrogenic, adipogenic, and osteogenic capacity during expansion are therefore a prerequisite for effective translation (Pei, 2017).

Cell-derived decellularized extracellular matrix (dECM) has emerged as an effective preconditioning substrate that improves MSC lineage-specific differentiation potential during *in vitro* expansion (Pei et al., 2022; Wang et al., 2025). A key determinant of dECM bioactivity is its composition: dECM deposited by immortalized stem cells outperforms that from unmodified counterparts in restoring MSC chondrogenic capacity, an advantage attributed to enrichment in basement membrane components—particularly the heparan sulfate (HS) proteoglycan perlecan (Wang et al., 2021a; Wang et al., 2021b). Perlecan is a multidomain proteoglycan integral to the structural and signaling architecture of basement membranes and pericellular matrices, where it modulates tissue homeostasis and disease pathogenesis (Arikawa-Hirasawa, 2022). Expanding adult nucleus pulposus cells on perlecan-enriched dECM from perlecan-overexpressing fetal cells significantly enhanced their chondrogenic potential, whereas dECM from perlecan-deficient cells failed to provide equivalent benefit (Wang et al., 2025), indicating perlecan as a key determinant of dECM preconditioning efficacy—yet the specific functional moiety responsible remains unresolved.

Beyond its structural role, the HS glycosaminoglycan chains attached to the N-terminal domain I of perlecan are active regulators of MSC fate. These chains serve as co-receptor reservoirs for growth factors—including fibroblast growth factor-2 (FGF-2) and bone morphogenetic protein-2 (BMP-2)—facilitating their presentation to cell-surface receptors and modulating downstream signaling (Hayes et al., 2022a; Smith et al., 2007; Decarlo et al., 2012). Enzymatic removal of cell-surface HS chains with heparinase III (HPA) enhanced endogenous perlecan production and upregulated chondrogenic gene expression in murine bone marrow-derived MSCs (BMSCs) (Chung et al., 2024), while domain I HS chains of perlecan independently promoted cell aggregation and chondrogenic differentiation of C3H10T1/2 progenitor cells (French et al., 2002). Together, these studies indicate perlecan-associated HS chains as active modulators of MSC fate—influencing chondrogenic commitment and the stemness state that modulates differentiation competence.

Building on these foundations, we focused on infrapatellar fat pad-derived stem cells (IPFSCs), which possess robust intrinsic chondrogenic potential and deposit dECM enriched in basement membrane components (Wang et al., 2021a; Wang et al., 2020). dECM from IPFSCs immortalized with SV40 large T antigen (SV40LT)—which bypasses replicative senescence by inactivating p53 and pRb (Sáenz Robles and Pipas, 2009)—exhibits markedly higher perlecan expression and confers superior chondrogenic enhancement relative to dECM from unmodified cells (Wang et al., 2021a; Wang et al., 2021b). We therefore used SV40LT-transduced IPFSCs to generate a perlecan-enriched dECM model (s-dE), alongside dECM from unmodified IPFSCs (dE) as a comparator. We hypothesized that dECM bioactivity is functionally dependent on perlecan-associated HS chains, and that their selective removal by HPA would reveal source-dependent roles for these chains in modulating stemness maintenance and multilineage differentiation of expanded IPFSCs. To test this, both dECM types were subjected to HPA treatment, yielding four dECM conditions—dE, dE(H), s-dE, and s-dE(H)—evaluated for effects on stemness and chondrogenic, adipogenic, and osteogenic differentiation capacity.

## 2 Materials and methods

### 2.1 Study design

This study evaluated the effects of dECM composition and HPA-mediated HS chain removal on the multilineage differentiation potential of adult IPFSCs, which display an intrinsic propensity for chondrogenic differentiation (Vahedi et al., 2021). Control cells were expanded on standard tissue culture plastic (TCP; PL group). Experimental cells were preconditioned on dE or s-dE; each substrate was tested with and without HPA treatment to ablate HS chains. This yielded five groups in total: PL, dE, dE(H), s-dE, and s-dE(H). All assays were performed with at least three technical replicates (n = 3) per group using cells pooled from six donors.

### 2.2 Cell culture and SV40LT transduction

Human IPFSCs were isolated from the infrapatellar fat pads of six de-identified young donors (four male, two female; mean age 22 years) presenting with acute meniscus or anterior cruciate ligament tears (Wang et al., 2025; Wang et al., 2019). The tissues were pooled and digested to obtain primary cells, which were expanded and cryopreserved at passage 3 (P3). Cells were maintained in complete growth medium [α-MEM supplemented with 10% fetal bovine serum (FBS), 100 U/mL penicillin, 100 μg/mL streptomycin, and 0.25 μg/mL fungizone] at 37 °C in a humidified incubator with 5% CO_2_ and 21% O_2_. Medium was replaced every 3 days.

For SV40LT transduction, P3 IPFSCs were thawed, expanded, and divided into two parallel groups: one for normal culture and one for SV40LT transduction. Transduction was performed by incubating cells for 24 h with the lentiviral expression vector (pRSC-EF1-SV40LT-E2A-Puro-wpre) in α-MEM containing 10% FBS and 1.5% protamine sulfate (MilliporeSigma, Germany) to improve transduction efficiency, as described previously (Wang et al., 2021a; Pei et al., 2023). Stably transduced cells were selected in 2 μg/mL puromycin (MilliporeSigma) for six consecutive days. Both normal and SV40LT-transduced cells were passaged in parallel under identical conditions. At P6, cells were used for dECM deposition (dE and s-dE, respectively). For preconditioning, P6 normal IPFSCs were seeded onto dE or s-dE at 3,000 cells/cm^2^ and cultured for approximately 10 days (single passage) to 95%–100% confluence, with standardized timing across all groups. Cells were harvested with type II collagenase from dECM substrates or with trypsin from PL, and then used for trilineage differentiation assays (chondrogenic, osteogenic, and adipogenic). P5–P6 cells were selected for these experiments as they retain robust proliferative and multilineage differentiation capacity relative to higher passages (Li and Pei, 2012; Wang et al., 2021a).

### 2.3 dECM fabrication

dECM was prepared following a previously validated protocol (Li and Pei, 2018). Briefly, TCP surfaces were coated with 0.2% gelatin (MilliporeSigma) at 37 °C for 1 h, then crosslinked by sequential treatment with 1% glutaraldehyde (MilliporeSigma) and 1 M ethanolamine (MilliporeSigma) for 30 min each, to stabilize the coating and quench residual reactive groups. P6 IPFSCs (transduced or non-transduced) were seeded at 6,000 cells/cm^2^ and cultured to confluence. Matrix deposition was stimulated by supplementing the medium with 250 μM L-ascorbic acid 2-phosphate (Wako Chemicals, Japan) for 8 days post-confluence. Cells were removed by incubation with 0.5% Triton X-100 (MilliporeSigma) containing 20 mM ammonium hydroxide (Sargent-Welch, United States) at 37 °C for 5 min. The resulting dECM was rinsed extensively with phosphate-buffered saline (PBS) and stored at 4 °C in PBS supplemented with 100 U/mL penicillin, 100 μg/mL streptomycin, and 0.25 μg/mL fungizone until use.

### 2.4 Enzymatic removal of HS chains from dECM

HPA (50 U; MilliporeSigma) was reconstituted in 50 mL of reaction buffer (20 mM Tris-HCl, 4 mM CaCl_2_, 0.1 mg/mL BSA, pH 7.5) and applied to dECM substrates (dE or s-dE) at 0.04 U/cm^2^ for 1 h at 37 °C. Control substrates were incubated with buffer alone under identical conditions. After digestion, substrates were rinsed twice with PBS and used immediately for cell expansion.

### 2.5 Immunofluorescence staining of dECM before and after decellularization

Pre- and post-decellularization matrix samples were blocked with 1% BSA in PBS for 1 h, then incubated for 2 h with primary antibodies: anti-human perlecan (1:100; Santa Cruz Biotechnology, United States), anti-3G10 (1:100; Amsbio, United States), and anti-10E4 (1:100; Amsbio, United States). 3G10 recognizes a neo-epitope exposed on HS stubs after heparinase cleavage; 10E4 binds intact, unmodified HS chains (David et al., 1992). After PBS washing, samples were incubated for 1 h with secondary antibodies: Alexa Fluor 488-conjugated anti-rat IgG (1:250; Invitrogen, United States) for perlecan; Alexa Fluor 594-conjugated anti-mouse IgG (1:200; Invitrogen, United States) for 3G10; and Alexa Fluor 594-conjugated anti-mouse IgM (1:100; Invitrogen, United States) for 10E4. Nuclei were counterstained with DAPI, and images were acquired on a Zeiss Axiovert 40 CFL fluorescence microscope.

### 2.6 Assessment of stemness gene expression and surface marker phenotype

After dECM expansion, stemness-associated gene expression was quantified by RT-qPCR (n = 3): *SOX2* (SRY-box transcription factor 2; Hs01053049_s1), *NANOG* (Hs02387400_g1), *NES* (Nestin; Hs04187831_g1), *MYC* (MYC proto-oncogene; Hs00153408_m1), *POU5F1* (POU class 5 homeobox 1; Hs04260367_gH), *KLF4* (Krüppel-like factor 4; Hs00358836_m1), *BMI1* (Hs00995536_m1), and *NOV* (nephroblastoma overexpressed; Hs00159631_m1). Relative expression was calculated by the 2^−ΔΔCT^ method and normalized to *GAPDH* (glyceraldehyde 3-phosphate dehydrogenase; Hs02758991_g1) as the endogenous reference. The SOX2 expression level in the PL group was used as the common calibrator and set to 1. All values, including other genes in the PL group and all genes in the other groups, are expressed as fold changes relative to this same calibrator. Therefore, only PL-group SOX2 equals 1, whereas other PL-group genes (e.g., NES, MYC, NOV) are not 1. Comparisons were made only within the same gene across different groups, not between different genes.

For surface marker profiling, confluent expanded cells (n = 3) were pre-blocked with 0.1% human IgG (Jackson ImmunoResearch Laboratories, United States) in PBS for 30 min at 4 °C, then stained for 30 min at 4 °C in the dark with fluorochrome-conjugated antibodies against CD73 (eBioscience, United States), CD90 (Miltenyi Biotec, United States), CD105 (Miltenyi Biotec, United States), CD146 (eBioscience, United States), and SSEA4 (BioLegend, United States). After PBS washing, data were acquired on a BD FACSCalibur™ flow cytometer and analyzed with FCS Express 7.0 Research Edition (*De Novo* Software).

### 2.7 Chondrogenic differentiation and assessment

Chondrogenic differentiation was induced in a three-dimensional pellet culture system. Aliquots of 3 × 10^5^ expanded cells were centrifuged at 1,200 rpm for 7 min in 15 mL polypropylene tubes. Pellets were pre-cultured in complete growth medium for 24 h to allow aggregation (day 0), then transferred to chondrogenic induction medium: high-glucose DMEM supplemented with 40 μg/mL L-proline, 100 nM dexamethasone (MilliporeSigma), 0.1 mM L-ascorbic acid 2-phosphate (Wako Chemicals), 100 U/mL penicillin, 100 μg/mL streptomycin, 1× ITS™ Premix (BD Biosciences, United States), and 10 ng/mL TGF-β3 (PeproTech, United States). Pellets were cultured for 21 days at 37 °C under hypoxic conditions (5% O_2_) with medium changes every 2–3 days.

Cells were harvested at day 0 (n = 3) and day 21 (n = 3) for RT-qPCR analysis of chondrogenic marker genes using TaqMan gene expression assays: *SOX9* (SRY-box transcription factor 9; Hs00165814_m1), *ACAN* (aggrecan; Hs00153936_m1), and *COL2A1* (type II collagen; Hs00156568_m1). *GAPDH* served as the endogenous reference.

For histological and immunohistochemical (IHC) assessment, representative pellets (n = 3) were fixed in 4% paraformaldehyde (PFA) at 4 °C for 30 min, dehydrated through a graded ethanol series, cleared in xylene, and paraffin-embedded. Sections (4 μm) were stained with Alcian blue (Ab) and Safranin O (SO) to visualize sulfated glycosaminoglycans (sGAGs). For IHC detection of type II collagen (Col2), sections were treated with 1% H_2_O_2_ for 10 min to quench endogenous peroxidase activity, then subjected to antigen retrieval with 2 mg/mL hyaluronidase (MilliporeSigma) at 37 °C for 30 min. Non-specific binding was blocked with 1% normal horse serum for 30 min. Sections were incubated with primary antibody against Col2 (II-II6B3; Developmental Studies Hybridoma Bank, United States; 1:500), then with biotinylated horse anti-mouse/rabbit IgG (H + L) secondary antibody (Vector Laboratories, United States). Signal was detected using the VECTASTAIN® ABC kit (Vector Laboratories) with 3,3′-diaminobenzidine (DAB; Vector Laboratories) as the chromogenic substrate.

### 2.8 Adipogenic differentiation and assessment

Following preconditioning on dECM substrates, cells were harvested and digested with trypsin. An aliquot of the digested cells was collected directly as day-0 samples. The remaining cells were replated on plastic. At approximately 95% confluence, complete growth medium was replaced with adipogenic induction medium: complete medium supplemented with 1 μM dexamethasone (MilliporeSigma), 0.5 mM isobutylmethylxanthine (IBMX; MilliporeSigma), 200 μM indomethacin (MilliporeSigma), and 10 μM insulin (BioVendor LLC, United States). Cells were maintained for 21 days at 37 °C in a normoxic incubator with 5% CO_2_.

After 21 days, cells (n = 3) were rinsed with PBS, fixed in 4% PFA for 30 min, and stained with freshly prepared 0.6% (w/v) Oil Red O (ORO; 60% isopropanol in distilled water) for 15 min with gentle agitation. Excess stain was removed by washing with distilled water, and images were captured on an Olympus IX51 inverted microscope. For semi-quantification, bound ORO was eluted with 100% isopropanol and absorbance measured at 510 nm on a microplate reader.

Adipogenic gene expression was assessed by RT-qPCR using TaqMan gene expression assays (n = 3) for *LPL* (lipoprotein lipase; Hs00173425_m1), *FABP4* (fatty acid-binding protein 4; Hs00609791_m1), *CEBPA* (CCAAT/enhancer-binding protein alpha; Hs00269972_s1), and *PPARG* (peroxisome proliferator-activated receptor gamma; Hs01115513_m1), with *GAPDH* as the endogenous reference.

### 2.9 Osteogenic differentiation and assessment

Following dECM preconditioning, cells were harvested and digested with trypsin. An aliquot of the digested cells was collected directly as day-0 samples. The remaining cells were replated on plastic. For osteogenic induction, 2 × 10^5^ expanded cells were seeded into T25 flasks, cultured to confluence, then switched to osteogenic induction medium: complete medium supplemented with 10 nM dexamethasone (MilliporeSigma), 10 mM β-glycerophosphate (MilliporeSigma), and 0.2 mM ascorbic acid 2-phosphate (Wako Chemicals). Cultures were maintained for 21 days at 37 °C in a normoxic incubator with 5% CO_2_; medium was changed every 3 days.

After 21 days, cells (n = 3) were fixed in 4% PFA for 30 min and stained with 2% Alizarin Red S (ARS) for 20–30 min with gentle agitation. Calcium deposits were imaged on an Olympus IX51 inverted microscope. For semi-quantification, bound ARS was extracted with 10% (v/v) acetic acid for 30 min, neutralized with 10% (v/v) ammonium hydroxide, and absorbance measured at 405 nm on a microplate reader.

Osteogenic gene expression was quantified by RT-qPCR using TaqMan gene expression assays (n = 3) for *RUNX2* (runt-related transcription factor 2; Hs00231692_m1), *BGLAP* (osteocalcin; Hs01587814_g1), *SP7* (osterix; Hs01866874_s1), and *ALPL* (tissue-nonspecific alkaline phosphatase; Hs01029144_m1), with *GAPDH* as the endogenous reference.

### 2.10 Statistical analysis

All comparisons among multiple groups were performed using one-way ANOVA followed by Tukey’s post hoc test. Data are presented as mean ± SD. Groups not sharing the same letter in figures differ significantly (*P* < 0.05, two-tailed). All analyses were performed in SPSS 20.0 (IBM).

## 3 Results

### 3.1 HPA-mediated removal of HS chains from perlecan in dECM

Successful decellularization was confirmed by the absence of cell nuclei in both dE and s-dE matrices (Figure 1). To verify HPA-mediated HS chain removal, pre- and post-decellularized samples were examined by immunofluorescence with two complementary antibodies. In untreated conditions, 10E4 (which recognizes intact HS chains) colocalized with perlecan in cells and dECM (Figure 1). After HPA treatment, 10E4 signal was markedly reduced in the dECM, indicating efficient HS chain cleavage; however, intense perinuclear 10E4 staining persisted in cells, consistent with intracellular HS chains undergoing biosynthesis in the Golgi apparatus and therefore inaccessible to HPA (Figure 1). Conversely, 3G10—which recognizes the neo-epitope on HS stubs exposed by heparinase cleavage—yielded strong signal in HPA-treated cells and in both dE (H) and s-dE (H) matrices, while remaining absent in untreated controls (Figure 1). Together, these data confirm that HPA selectively ablates HS chains from matrix-resident perlecan without disrupting the core protein.

**FIGURE 1:**
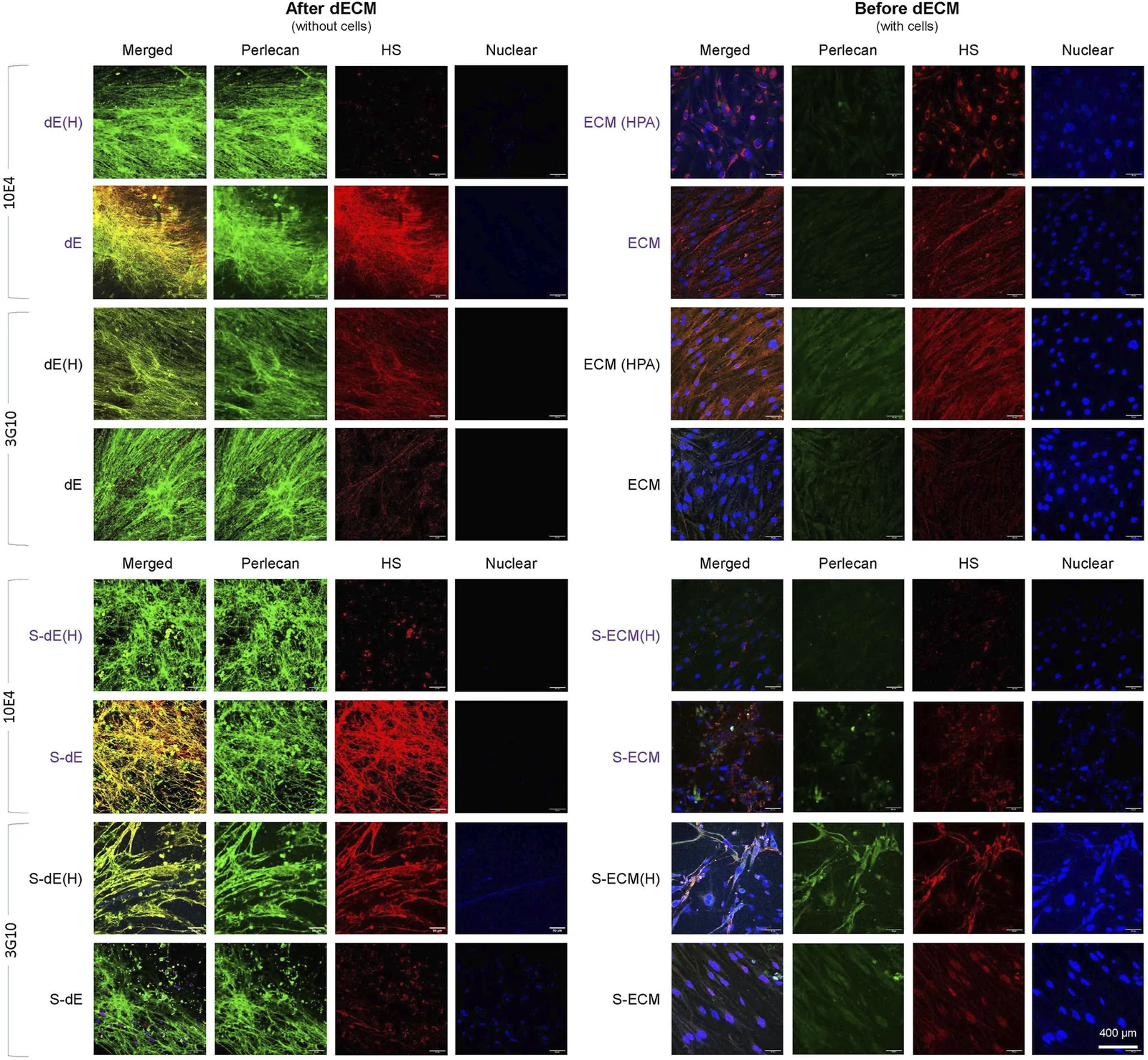
Immunofluorescence characterization of perlecan and heparan sulfate (HS) before and after decellularization. Representative images show perlecan (green), intact HS chains (10E4, red), and HPA-generated HS stubs (3G10, red); nuclei were counterstained with DAPI (blue). The figure is divided into two main blocks: the left block shows matrices after decellularization (After), and the right block shows matrices before decellularization (Before). Within each block, the upper half shows ECMs deposited by normal cells, and the lower half shows ECMs deposited by SV40LT-induced cells. In each ECM type, 10E4 is shown above 3G10, and +HPA is shown above –HPA within each channel. Scale bars: 400 μm.

### 3.2 Effects of HPA-treated dECM on stemness gene expression and surface marker profile of adult IPFSCs

Adult IPFSCs expanded on all four dECM substrates (dE, dE(H), s-dE, and s-dE(H)) displayed a smaller, more spindle-shaped morphology with enhanced refractility relative to cells grown on PL, as observed by phase-contrast microscopy (Figure 2A). RT-qPCR analysis revealed that expansion on dE or s-dE significantly modulated the expression of stemness-associated genes compared with the PL group. The upregulated genes included *SOX2* (dE: 2.142 ± 0.548; s-dE: 10.609 ± 1.080; PL: 1.002 ± 0.085), *NANOG* (dE: 11.468 ± 0.306; s-dE: 48.762 ± 4.770; PL: 6.674 ± 1.233), and *NES* (dE: 563.983 ± 1.949; s-dE: 867.773 ± 9.769; PL: 236.150 ± 6.235). In contrast, the downregulated genes were *MYC* (dE: 696.036 ± 23.256; s-dE: 676.946 ± 4.853; PL: 1,657.124 ± 26.031), *NOV* (dE: 140.191 ± 5.323; s-dE: 98.315 ± 0.282; PL: 2,514.556 ± 112.088), *BMI1* (dE: 291.377 ± 5.483; s-dE: 233.422 ± 9.460; PL: 595.616 ± 30.040), and *KLF4* (dE: 244.661 ± 35.670; s-dE: 193.175 ± 11.408; PL: 746.286 ± 19.435) (Figure 2B; *P < 0.05*). Cells expanded on s-dE displayed even greater upregulation of *SOX2*, *NANOG*, and *NES* than those on dE. HPA treatment attenuated *SOX2* (dE(H): 0.543 ± 0.095; s-dE(H): 0.550 ± 0.043), *NANOG* (dE(H): 6.101 ± 0.248; s-dE(H): 7.083 ± 0.471), *NES* (dE(H): 408.590 ± 0.382; s-dE(H): 357.973 ± 5.664) in both groups while upregulating *NOV* (dE(H): 499.481 ± 1.454; s-dE(H): 156.963 ± 9.403) in both HPA-treated groups (*P < 0.05*); *KLF4* was selectively attenuated in the s-dE(H) group, but not in dE(H) (dE(H): 299.687 ± 14.720; s-dE(H): 123.302 ± 2.745); and *MYC* exhibited divergent responses depending on the dECM source—elevated in dE(H) (1,000.552 ± 92.489) relative to dE but further reduced in s-dE(H) (618.732 ± 19.807) relative to s-dE (Figure 2B; *P < 0.05*). Expression of *POU5F1* showed the highest expression in the s-dE group (152.571 ± 6.850), which was significantly reduced by HPA treatment in s-dE(H) (67.874 ± 1.421), while dE-expanded cells (38.010 ± 2.160) expressed lower levels than PL (108.027 ± 5.671) and dE(H) (43.504 ± 0.594) showed a modest partial recovery (Figure 2B; *P < 0.05*).

**FIGURE 2:**
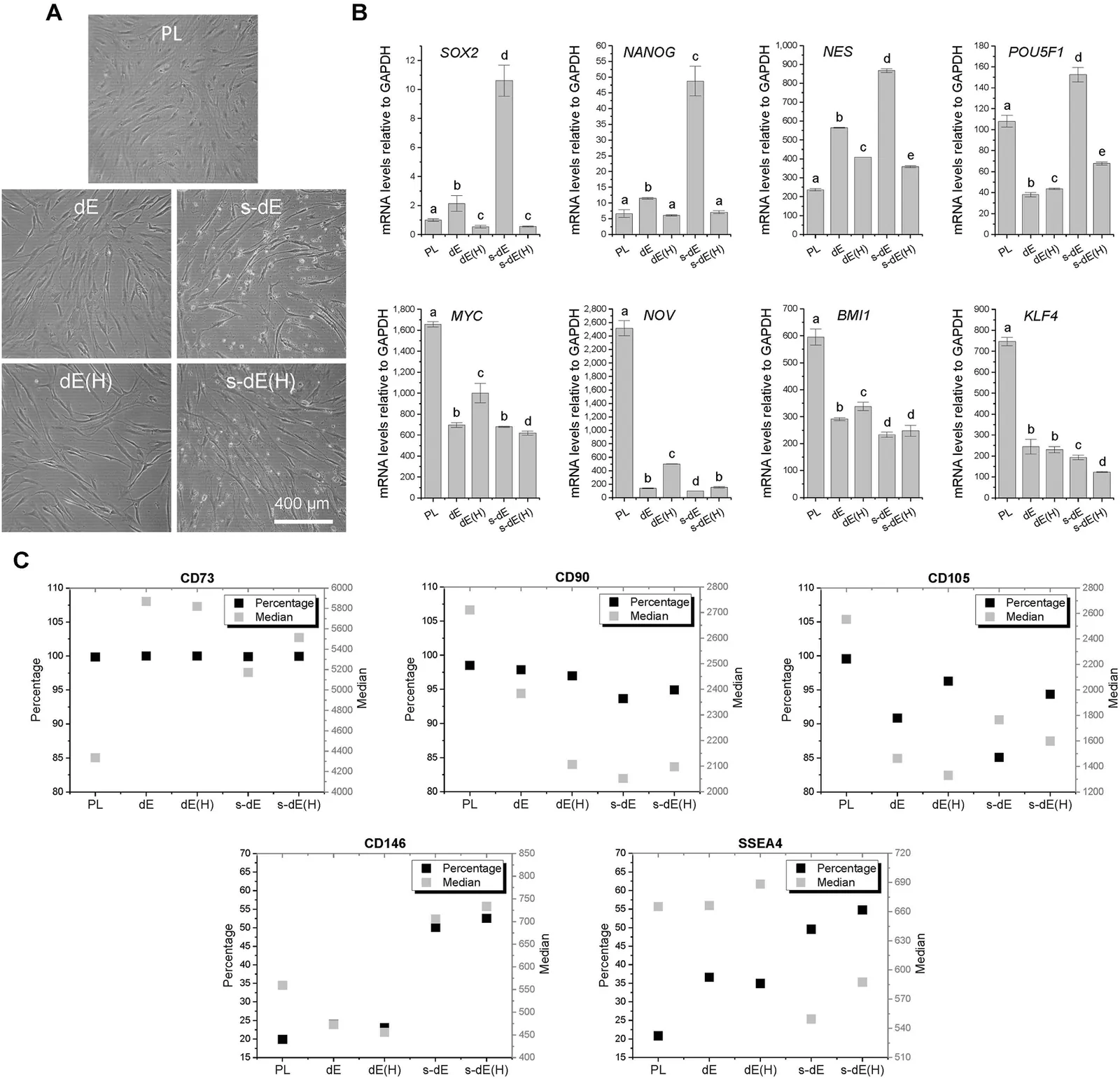
Effects of dECM preconditioning on IPFSC morphology, stemness gene expression and surface marker phenotype. **(A)** Phase-contrast micrographs of P6 IPFSCs expanded on PL, dE, dE(H), s-dE, and s-dE(H). Scale bars: 400 μm. **(B)** RT-qPCR analysis of stemness-related gene expression (*SOX2*, *NANOG*, *POU5F1*, *NES*, *MYC*, *NOV*, *BMI1*, and *KLF4*) in expanded cells. Expression was normalized to *GAPDH* and calculated by the 2^−ΔΔCT^ method. Data represent mean ± SD. Different letters denote statistically significant differences (*P < 0.05*). **(C)** Representative flow cytometric analysis of MSC surface markers (CD73, CD90, CD105, CD146, and SSEA4) in expanded IPFSCs. Both the percentage of positive cells (left axis) and the median fluorescence intensity (MFI; right axis) are shown.

Compared with PL, expansion on dE or s-dE reduced the percentage and median fluorescence intensity (MFI) of CD90 and CD105 while elevating CD146 and SSEA4 positivity; dE and s-dE expansion also increased the MFI of CD73 (Figure 2C; Supplementary Figure S1). HPA treatment increased the percentage of CD105-positive cells in both dE(H) and s-dE(H), whereas CD73 positivity remained near 100% across all groups. The effect of HS chain removal on CD90, CD146, and SSEA4 was substrate-dependent: in the dE model, HPA reduced SSEA4 positivity with only marginal effects on CD90 and negligible effects on CD146; in the s-dE model, HPA upregulated all three, with s-dE(H)-preconditioned cells exhibiting the highest CD146 and SSEA4 percentages across all groups (Figure 2C; Supplementary Figure S1).

### 3.3 Differential modulation of IPFSC chondrogenic capacity by HPA-treated dECM

Chondrogenic potential was assessed after 21 days of pellet culture by RT-qPCR for *SOX9*, *ACAN*, and *COL2A1* expression, alongside Ab and SO staining for sGAGs and immunohistochemical analysis of Col2. At day 0, expansion on dE or s-dE increased *COL2A1* expression (dE: 10.272 ± 0.323; s-dE: 5.924 ± 2.317; PL: 1.000 ± 0.000; *P < 0.05*) while decreasing *ACAN* (dE: 0.005 ± <0.001; s-dE: 0.003 ± <0.001; PL: 1.000 ± 0.023; *P < 0.05*) relative to PL; however, *SOX9* was unchanged at day 0 (Figure 3B). After 21 days of chondrogenic induction, cells preconditioned on dE or s-dE formed markedly larger pellets (Figure 3A) and expressed significantly higher *SOX9* (dE: 8.465 ± 0.293; s-dE: 7.720 ± 0.044; PL: 7.079 ± 0.313), *ACAN* (dE: 7.319 ± 0.254; s-dE: 8.121 ± 0.379; PL: 4.132 ± 0.133), and *COL2A1* (dE: 51,352.216 ± 1,076.990; s-dE: 49,522.193 ± 1,436.681; PL: 8,202.145 ± 319.994) than PL-expanded cells (Figure 3B; *P < 0.05*). Histological staining corroborated these results, with dE and s-dE pellets showing substantially greater Ab- and SO-positive staining, consistent with enhanced sGAG accumulation (Figure 3A).

**FIGURE 3:**
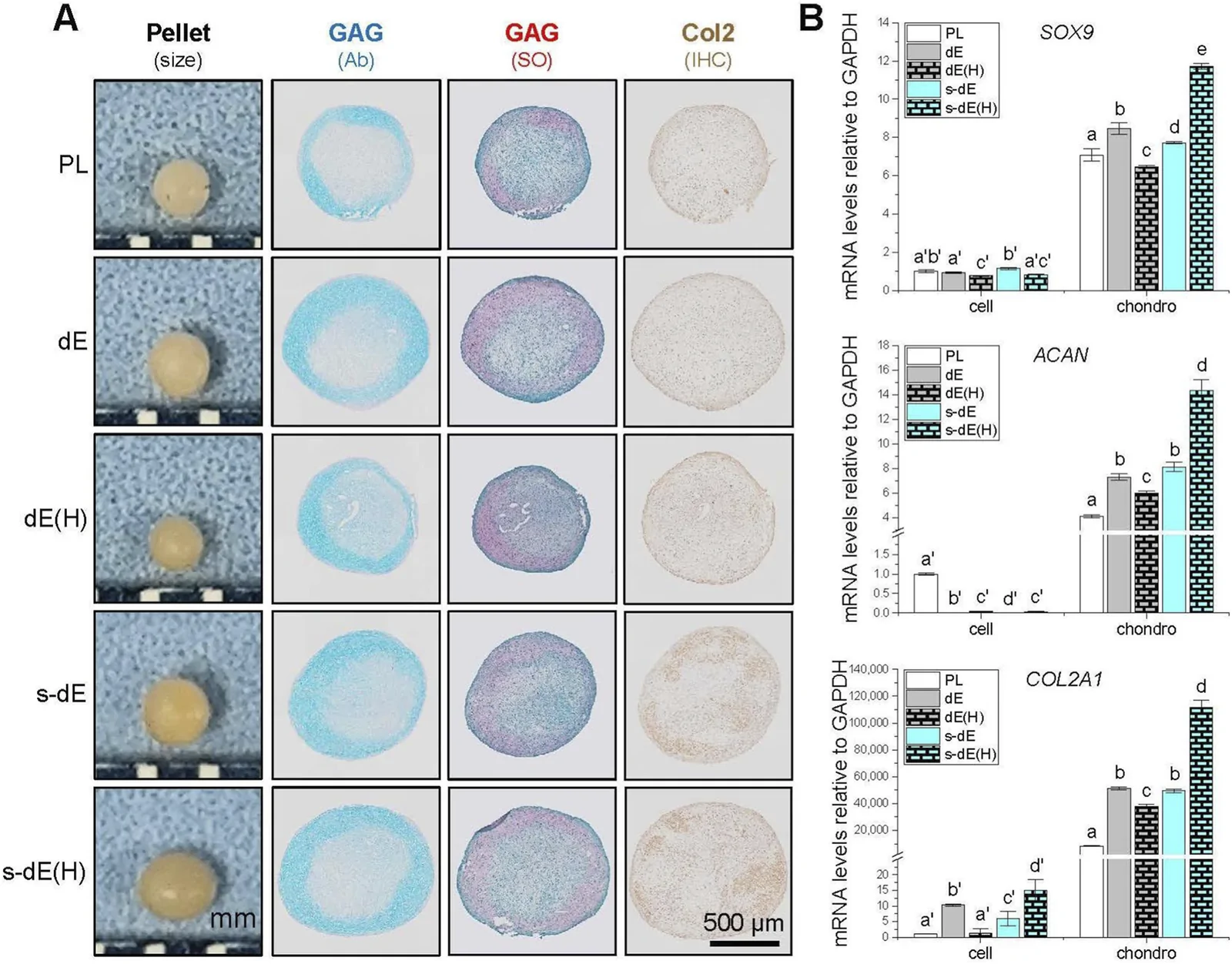
Effects of dECM preconditioning on the chondrogenic potential of P6 IPFSCs. **(A)** Representative images of day 21 chondrogenic pellets. From left to right: gross morphology with scale bar (mm); Alcian blue (Ab) staining for sulfated glycosaminoglycans (sGAGs); Safranin O (SO) staining for sGAGs; and immunohistochemical (IHC) staining for type II collagen (Col2). Scale bars: 500 μm. **(B)** RT-qPCR analysis of chondrogenic marker gene expression (*SOX9*, *ACAN*, *COL2A1*) at day 0 (cell; prior to induction) and day 21 (chondro; after induction). Expression was normalized to *GAPDH*. Data represent mean ± SD. Different letters within each time point denote statistically significant differences (*P < 0.05*); primed letters (a′, b′) indicate comparisons within the day 0 group.

HPA treatment produced divergent effects depending on the dECM source. At day 0, both dE(H) and s-dE(H) groups showed downregulated *SOX9* (dE: 0.918 ± 0.043 vs. dE(H): 0.759 ± 0.008; s-dE: 1.145 ± 0.052 vs. s-dE(H): 0.821 ± 0.021; *P < 0.05*) but upregulated *ACAN* (dE(H): 0.041 ± 0.002; s-dE(H): 0.034 ± 0.003; *P < 0.05*) relative to their untreated counterparts—with *COL2A1* reduced in dE(H) (1.265 ± 1.357; *P < 0.05*) but elevated in s-dE(H) (14.954 ± 3.535; *P < 0.05*). Following chondrogenic induction, these source-dependent divergences were amplified: dE(H)-preconditioned cells showed significantly reduced *SOX9* (dE(H): 6.458 ± 0.075), *ACAN* (dE(H): 6.009 ± 0.166), and *COL2A1* (dE(H): 38,001.631 ± 1,339.186) versus dE, whereas s-dE(H)-preconditioned cells showed markedly elevated expression of all three (s-dE(H): *SOX9*: 11.713 ± 0.164; *ACAN*: 14.310 ± 0.891; *COL2A1*: 111,695.573 ± 5,567.585) versus s-dE (Figure 3B; *P < 0.05*). These transcriptional differences were paralleled by histological findings: the dE(H) group showed reduced Ab and SO staining, while the s-dE(H) group exhibited the most extensive sGAG deposition and the largest pellet size of all dECM conditions (Figure 3A).

### 3.4 Enhancement of IPFSC adipogenic potential by HPA-treated dECM

Adipogenic potential was evaluated after 21 days of induction by RT-qPCR for *LPL*, *FABP4*, *CEBPA*, and *PPARG*, and ORO staining for intracellular lipid accumulation. Despite elevated *CEBPA* (dE: 6.509 ± 0.257; s-dE: 7.435 ± 0.253; PL: 1.000 ± 0.037; *P < 0.05*) and reduced *FABP4* (dE: 0.183 ± 0.037; s-dE: 0.420 ± 0.028; PL: 1.026 ± 0.325; *P < 0.05*) expression at day 0, both dE and s-dE groups showed significantly lower expression of all four adipogenic markers after induction compared to PL (Figure 4C; *P < 0.05*), consistent with markedly reduced lipid droplet accumulation by ORO staining in both groups (absorbance values: dE: 0.214 ± 0.005; s-dE: 0.229 ± 0.004; PL: 0.431 ± 0.008; Figures 4A,B; *P < 0.05*). Notably, the s-dE group displayed significantly higher expression of *LPL* (s-dE: 31,074.779 ± 760.951 vs. dE: 23,792.795 ± 96.533), *FABP4* (s-dE: 0.420 ± 0.028 vs. dE: 0.183 ± 0.037), and *CEBPA* (s-dE: 553.113 ± 7.587 vs. dE: 381.940 ± 4.772) than the dE group after induction (Figure 4C; *P < 0.05*). In contrast, *PPARG* was lower in the s-dE group compared with the dE group (69.369 ± 0.639 vs. 74.649 ± 2.430) (Figure 4C; *P < 0.05*), suggesting a selective regulatory pattern among adipogenic markers.

**FIGURE 4:**
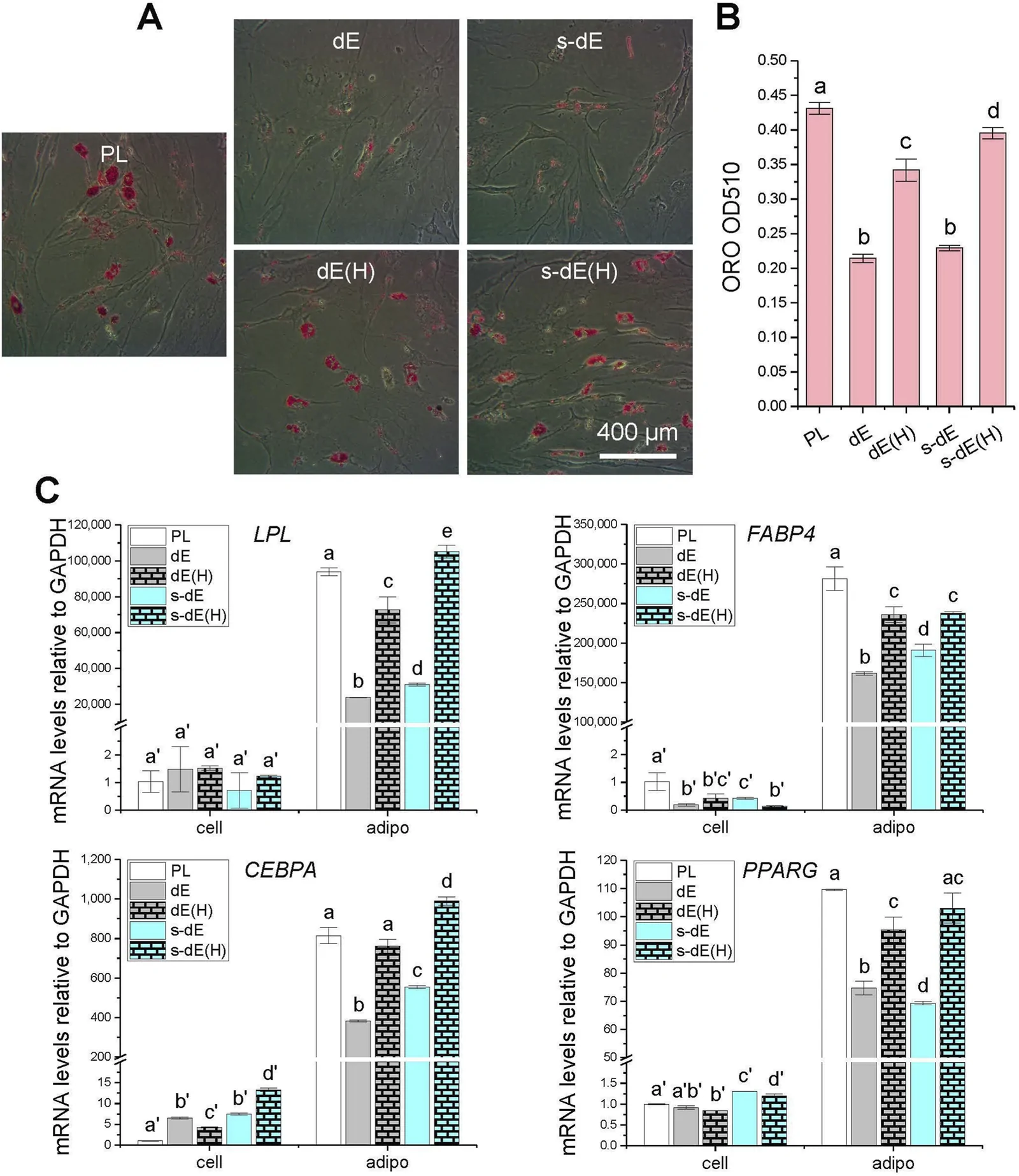
Effects of dECM preconditioning on the adipogenic potential of P6 IPFSCs. **(A)** Representative Oil Red O (ORO) staining for intracellular lipid droplets following 21 days of adipogenic induction. Scale bars: 400 μm. **(B)** Semi-quantification of ORO staining by absorbance at 510 nm. Data represent mean ± SD. Different letters denote statistically significant differences (*P < 0.05*). **(C)** RT-qPCR analysis of adipogenic marker gene expression (*LPL*, *FABP4*, *CEBPA*, *PPARG*) at day 0 (cell) and day 21 (adipo). Expression was normalized to *GAPDH*. Data represent mean ± SD. Different letters within each time point denote statistically significant differences (*P < 0.05*); primed letters indicate comparisons within the day 0 group.

HPA treatment substantially enhanced adipogenic responsiveness in both groups. dE(H)- and s-dE(H)-preconditioned cells exhibited pronounced increases in lipid droplet accumulation by ORO staining (absorbance values: dE(H): 0.342 ± 0.016; s-dE(H): 0.395 ± 0.007; Figures 4A,B; *P < 0.05*), supported transcriptionally by significant upregulation of *LPL* (dE(H): 72,738.920 ± 7,224.080; s-dE(H): 105,194.291 ± 3,661.168), *FABP4* (dE(H): 235,945.190 ± 10,265.078; s-dE(H): 237,806.626 ± 1,632.430), *CEBPA* (dE(H): 760.703 ± 35.155; s-dE(H): 989.707 ± 20.563), and *PPARG* (dE(H): 95.404 ± 4.448; s-dE(H): 103.092 ± 5.412) upon induction relative to their untreated counterparts (Figure 4C; *P < 0.05*).

### 3.5 s-dE(H) selectively promotes osteogenic matrix mineralization in IPFSCs

Osteogenic potential was assessed after 21 days by RT-qPCR for *RUNX2*, *BGLAP*, *SP7*, and *ALPL* and ARS staining for matrix mineralization. Mineralized nodule formation was observed exclusively in the s-dE(H) group, confirmed by semi-quantitative ARS absorbance (absorbance values: s-dE(H): 0.766 ± 0.029; Figures 5A,B; *P < 0.05*). Interestingly, cells expanded on dE or s-dE exhibited lower *ALPL* (dE: 0.698 ± 0.072; s-dE: 0.674 ± 0.002; PL: 1.000 ± 0.035), but higher *RUNX2* (dE: 1.509 ± 0.038; s-dE: 1.420 ± 0.023; PL: 1.000 ± 0.040) and *SP7* (dE: 2.283 ± 0.026; s-dE: 8.883 ± 0.045; PL: 1.008 ± 0.176) levels relative to PL (Figure 5C; *P < 0.05*). After 21 days of osteogenic induction, dE and s-dE groups both showed elevated *ALPL* (dE: 555.493 ± 6.377; s-dE: 538.131 ± 3.565; PL: 517.062 ± 11.498; *P < 0.05*) and *RUNX2* (dE: 7.669 ± 0.834; s-dE: 10.010 ± 0.146; PL: 5.452 ± 0.434; *P < 0.05*) levels compared to PL; however, the s-dE group showed reduced osteogenic responsiveness, with downregulation of *BGLAP* and *SP7* over time (Figure 5C).

**FIGURE 5:**
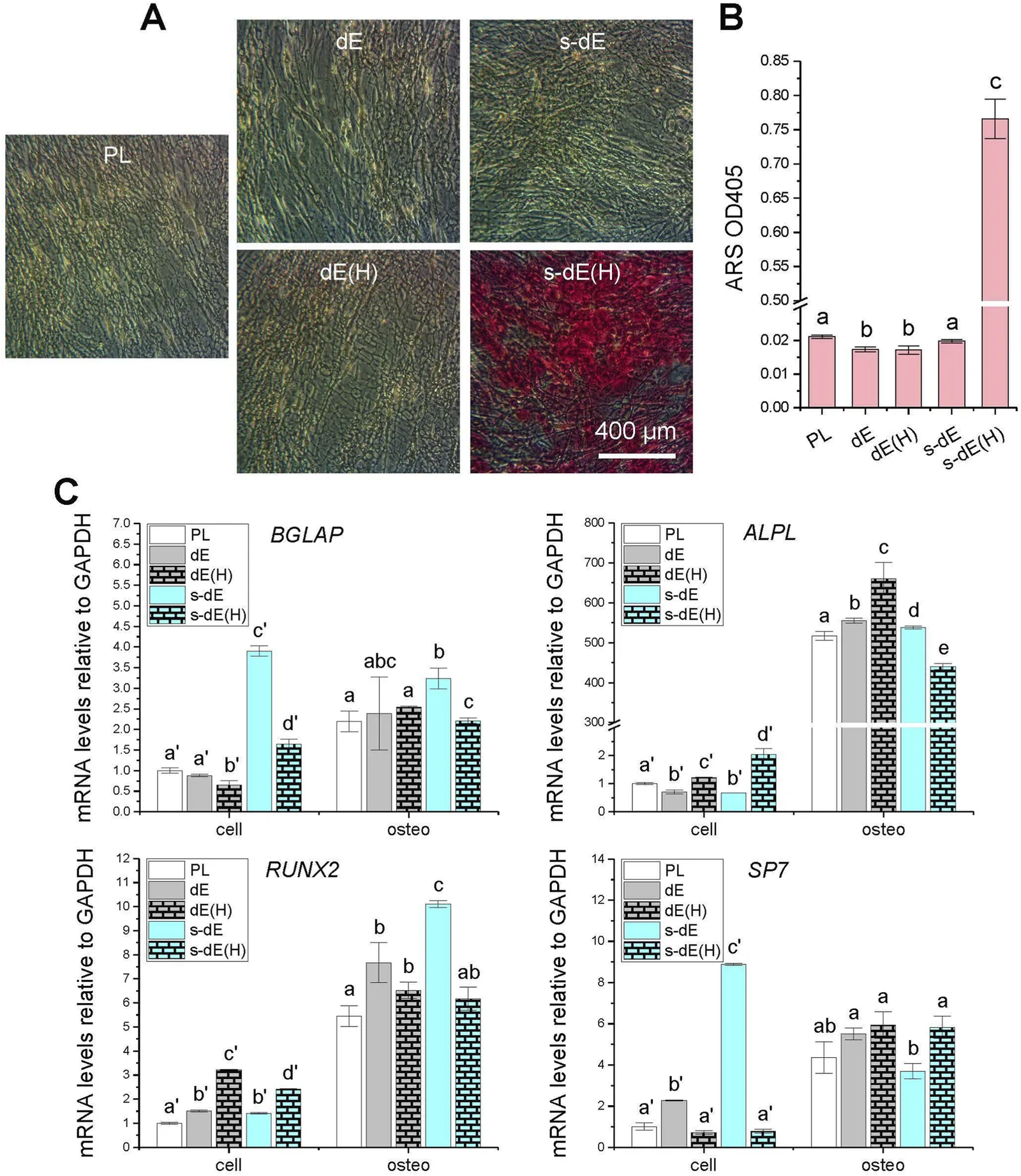
Effects of dECM preconditioning on the osteogenic potential of P6 IPFSCs. **(A)** Representative Alizarin Red S (ARS) staining for calcium deposits following 21 days of osteogenic induction. Scale bars: 400 μm. **(B)** Semi-quantification of ARS staining by absorbance at 405 nm. Data represent mean ± SD. Different letters denote statistically significant differences (*P < 0.05*). **(C)** RT-qPCR analysis of osteogenic marker gene expression (*BGLAP*, *ALPL*, *RUNX2*, *SP7*) at day 0 (cell) and day 21 (osteo). Expression was normalized to *GAPDH*. Data represent mean ± SD. Different letters within each time point denote statistically significant differences (*P < 0.05*); primed letters indicate comparisons within the day 0 group.

At day 0, both dE(H) and s-dE(H) showed downregulation of *BGLAP* (dE: 0.879 ± 0.030 vs. dE(H): 0.648 ± 0.114; s-dE: 3.902 ± 0.126 vs. s-dE(H): 1.646 ± 0.122; *P < 0.05*) and *SP7* (dE(H): 0.719 ± 0.086; s-dE(H): 0.791 ± 0.094; *P < 0.05*) concurrent with upregulation of *RUNX2* (dE(H): 3.214 ± 0.026; s-dE(H): 2.413 ± 0.027; *P < 0.05*) and *ALPL* (dE(H): 1.241 ± 0.013; s-dE(H): 2.050 ± 0.209; *P < 0.05*) relative to their untreated counterparts. After 21 days of induction, *RUNX2* trended downward in both HPA-treated groups. However, *SP7* was selectively elevated in s-dE(H) versus s-dE (s-dE: 3.697 ± 0.370 vs. s-dE(H): 5.833 ± 0.534), while *ALPL* was higher in dE(H) (660.324 ± 40.350) than dE (Figure 5C; *P < 0.05*). The exclusive osteogenic mineralization of s-dE(H)-preconditioned cells thus reflects a dissociation between transcriptional regulation and matrix mineralization, likely attributable to a temporal offset between peak gene expression and nodule formation.

## 4 Discussion

This study examined how HS chains on domain I of perlecan within the dECM regulate stemness and multilineage differentiation of adult IPFSCs. By applying HPA to selectively ablate HS chains from dE or s-dE, we isolated the contribution of matrix-resident HS chains from those on the cell surface—contrasting with conventional strategies that target cell-surface HS directly (Chung et al., 2024). Two matrix models were employed—dE and s-dE—both enriched in perlecan, with s-dE displaying a higher abundance of basement membrane components. This compositional distinction is consistent with the tumor-like phenotype acquired by SV40LT-mediated immortalization (Pez et al., 2019; Meng et al., 2025), and aligns with the well-established upregulation of perlecan in specific malignant versus normal tissues (Mishra et al., 2011). The findings reveal that the functional consequences of HS chain removal are modulated by the cellular origin of the deposited matrix, providing new insight into how extracellular perlecan HS architecture shapes stem cell fate.

HPA selectively cleaved perlecan-associated HS chains in the dECM without disrupting the core protein, as confirmed by loss of 10E4 signal (intact HS) and gain of 3G10 signal (HS stubs) in treated matrices. Notably, strong perinuclear 10E4 immunoreactivity persisted in cells after HPA treatment. This is explained by the membrane impermeability of HPA, which confines its activity to extracellular HS and cannot access nascent chains undergoing biosynthesis in the Golgi apparatus (Esko and Lindahl, 2001; Esko and Selleck, 2002); these intracellular chains become detectable only after cell permeabilization during immunostaining. These results confirm that the experimental design achieved selective ablation of matrix-resident HS while preserving intracellular pools, providing a clean platform for interrogating the role of extracellular perlecan HS chains in stem cell regulation.

Expansion on dE or s-dE upregulated the stemness-associated genes *SOX2*, *NANOG*, and *NES* in P6 IPFSCs relative to the PL control, consistent with prior observations in P15 IPFSCs (Wang et al., 2021a; Wang et al., 2021b). However, in contrast to P15 cells where *MYC*, *NOV*, and *BMI1* were upregulated by dECM expansion, P6 IPFSCs showed reduced expression of these genes. This passage-dependent divergence likely reflects the lower baseline senescence of P6 cells relative to P15 cells; as replicative senescence accumulates during *in vitro* expansion (Ogrodnik, 2021), early-passage cells may respond differently to the same matrix cues. An analogous maturation-state-dependent effect has been described for fetal versus adult cells preconditioned on adult cell-derived dECM (Li et al., 2015). HPA treatment of both dECM types attenuated *SOX2*, *NANOG*, and *NES* expression, consistent with depletion of HS-bound FGF ligands from the matrix. FGF signaling is a known driver of *SOX2* (Mansukhani et al., 2005), *NANOG* (Fojtík et al., 2025), and *NES* (Chen et al., 2010) expression, and perlecan HS chains serve as an essential reservoir for FGF-2 within the pericellular matrix (Hayes et al., 2022a); their removal by HPA therefore disrupts paracrine FGF signaling and suppresses stemness gene expression. The source-dependent divergence in *MYC* regulation (elevated in dE(H) but reduced in s-dE(H)) and the selective attenuation of *KLF4* only in s-dE(H) further indicate that the HS chains of the two perlecan pools differ in their capacity to sustain distinct transcriptional programs.

Expansion on dE or s-dE reduced the percentage and MFI of CD90 and CD105 while elevating SSEA4 positivity, consistent with prior observations in P15 IPFSCs (Wang et al., 2021a; Wang et al., 2021b). HPA treatment diverged the two models: in dE, the percentages of SSEA4, CD90, and CD146 were all marginally reduced; in s-dE, all three were paradoxically elevated, with s-dE(H)-preconditioned cells exhibiting the highest percentages of CD146 and SSEA4 across all groups. This substrate-dependent divergence in surface marker response parallels the opposing differentiation outcomes and further underscores the functional distinction between normal and immortalized IPFSC-derived perlecan HS chains. CD146 (also known as MUC-18/Mel-CAM) mediates signaling events that regulate proliferation and differentiation (Paduano et al., 2016), and CD146+ chondroprogenitors express markedly higher *SOX9*, *ACAN*, and *COL2A1* than CD146− cells (Vinod et al., 2021). Likewise, SSEA4+ progenitors possess potent osteogenic capacity (Li et al., 2014), which may account for the exclusive mineralized nodule formation in the s-dE(H) group. The enrichment of these progenitor subpopulations by s-dE(H) preconditioning thus provides a plausible surface-marker basis for the lineage-specific differentiation outcomes observed.

An apparent paradox emerges from these findings: in the s-dE(H) group, HPA treatment reduced the stemness-associated genes *SOX2*, *NANOG*, and *NES*, yet this same treatment markedly enhanced chondrogenic and osteogenic differentiation (detailed below). We propose that these two outcomes reflect distinct components of the perlecan HS reservoir rather than a single, unified response. Loss of the stemness signature was common to both dE(H) and s-dE(H) and likely reflects a shared mechanism—depletion of HS-sequestered FGF-2 that otherwise sustains a self-renewing, less-committed progenitor state. In contrast, the enhancement of differentiation capacity was restricted to s-dE(H) and implicates a distinct, source-specific mechanism: HS chains derived from immortalized-cell perlecan may normally mask, rather than present, FGF receptor-activating epitopes (elaborated below), such that their removal exposes latent lineage-promoting signaling. Under this model, reduced stemness gene expression does not indicate diminished differentiation potential; rather, exit from a stemness-associated transcriptional program in s-dE(H) coincides with, and may even be permissive for, entry into lineage-specific differentiation programs once these structurally distinct HS chains are removed.

The differential effects of HS chain removal on chondrogenic and osteogenic differentiation point to fundamental structural differences in perlecan HS chains between normal and SV40LT-immortalized IPFSCs. This source-dependent heterogeneity is not without precedent: cellular transformation broadly alters perlecan expression and HS chain characteristics, as illustrated by substantially higher perlecan levels in highly metastatic versus non-metastatic murine melanoma variants (Cohen et al., 1994). Perlecan from normal JB6 epidermal cells carries exclusively HS, whereas perlecan from the transformed RT101 line bears both HS and chondroitin sulfate (CS) with reduced HS sulfation; despite these differences, both variants bind FGF-1, -2, -4, and -7 with similar affinity (Tapanadechopone et al., 2001). More critically, perlecan cellular origin determines HS substructure in ways that profoundly affect FGF receptor activation: colon carcinoma-derived perlecan bound FGF-2 but failed to activate FGF receptors on BaF3 cells unless its HS chains were removed by HPA (Knox et al., 2002). Together, these findings hint that s-dE-derived perlecan HS chains may be functionally restricted for receptor activation, and that their removal by HPA unmasks the signaling potential of the core protein or exposes co-presented CS chains.

These findings are interpretable within the framework above. At day 0, stable *SOX9* expression indicated no precocious chondrogenic commitment; the observed suppression of *ACAN* and promotion of *COL2A1* at this stage likely reflects early biphasic transcriptional reprogramming rather than committed differentiation, consistent with prior P15 IPFSC data (Wang et al., 2021a; Wang et al., 2021b). Aggrecan is the principal load-bearing proteoglycan of cartilage ECM (Yan et al., 2026). Its dynamic modulation—downregulation during dECM expansion, then upregulation upon induction—suggests matrix priming initiates chondrogenic reprogramming without driving terminal commitment. After 21 days, dECM preconditioning substantially enhanced *SOX9*, *ACAN*, and *COL2A1* expression and sGAG deposition relative to PL. HPA then diverged the two groups: in dE, where HS chains normally sequester and present FGF-2, their removal eliminated this reservoir and impaired chondrogenic signaling, significantly reducing *SOX9*, *ACAN*, and *COL2A1*; in s-dE, where HS chains may be structurally incompetent for FGF receptor activation (analogous to Knox et al. (Knox et al., 2002)), their removal paradoxically liberated FGF-dependent signaling and amplified chondrogenic output. FGF-2 is a well-established positive regulator of chondrogenic commitment in MSCs (Pizzute et al., 2016; Qiu et al., 2026), and FGF-2 supplementation during dECM preconditioning amplifies chondrogenic capacity in synovium-derived stem cells (Li and Pei, 2011). To critically test this model, a parallel analysis of FGF-2 levels released from dECM prior to and following HPA digestion, alongside a time-course profiling of FGF-2 during cell expansion on dE versus s-dE, will be essential. Such investigations would help clarify whether the opposing chondrogenic outcomes are modulated by differential HS-mediated FGF-2 bioavailability and signaling, and represent a logical next direction for our ongoing work. Furthermore, to more fully understand the mechanistic basis for the divergent effects of dE and s-dE, it would be informative to compare the spectrum and abundance of HS binding growth factors, including FGF-2 and BMP-2, produced by normal versus SV40LT-transduced IPFSCs during matrix deposition. Differences in the secretory profiles of these two cell types could influence the growth factor repertoire sequestered by HS chains and may contribute to the distinct chondrogenic responses observed. Looking forward, it will be important to employ FGF receptor (FGFR)-blocking or neutralizing antibodies during cell expansion on dECM substrates to indicate whether the enhanced chondrogenic potential in s-dE(H)-preconditioned cell is causally dependent on FGFR signaling. Finally, a contribution from CS chains—potentially more abundant in immortalized-cell perlecan after HS removal—cannot be excluded, as CS is known to support cartilage matrix production (Brito et al., 2023).

HPA uniformly enhanced adipogenic potential regardless of dECM source, with increased lipid droplet accumulation and upregulation of *LPL*, *FABP4*, *CEBPA*, and *PPARG* in both dE(H) and s-dE(H). This convergent response is consistent with the established anti-adipogenic role of perlecan: its overexpression in adult IPFSCs suppresses lipid droplet formation, whereas genetic ablation enhances adipogenic differentiation (Wang et al., 2025). HPA-mediated cleavage of HS chains attenuates the growth factor-sequestering capacity of matrix-resident perlecan, relieving suppression of the adipogenic program. That this enhancement occurred in both dECM contexts despite their differing HS architectures suggests the anti-adipogenic function of perlecan is mediated primarily through its core protein or overall proteoglycan density, rather than HS chain-specific growth factor sequestration.

Osteogenic findings paralleled the chondrogenic pattern: only s-dE(H)-preconditioned cells formed abundant mineralized nodules at day 21, while all other groups showed minimal mineralization. This exclusive response is consistent with the elevated SSEA4 and CD146 percentages in s-dE(H)-preconditioned cells, given the documented osteogenic potency of SSEA4+ (Li et al., 2014) and CD146+ (Paduano et al., 2016) progenitors. The underlying mechanism likely parallels chondrogenesis: HS chain removal liberates FGF receptor-activating capacity from s-dE-derived perlecan, and brief FGF-2 exposure is sufficient to prime osteoprogenitors for robust mineralization (Sexton et al., 2024). Notably, the GAG chains on perlecan domain I also bind BMPs—key osteogenic morphogens—in addition to FGF-2 (Hayes et al., 2022b). Recent work has shown that a synthetic 6-O-sulfated HS glycopolymer enhances BMP receptor expression without altering BMP-2 ligand levels, thereby promoting BMSC mineralization more effectively than conventional HS—highlighting that the sulfation code of HS dictates its biological output (Xu et al., 2026). Furthermore, enzymatic removal of cell-surface HS has been reported to potentiate BMP signaling and canonical Wnt activity, thereby enhancing osteogenic differentiation (Manton et al., 2007). Collectively, these observations indicate that the strikingly enhanced mineralization observed exclusively in the s-dE(H) group may arise from one or more of the following: upregulated BMP receptor expression, potentiation of Wnt signaling, or transient release of matrix-sequestered BMPs during preconditioning (Lysdahl et al., 2014), each of which could collectively amplify the subsequent osteogenic response. While direct structural characterization of HS composition was not performed here, the functional divergence between the two matrices strongly supports the need for future glycomic analyses to define the molecular basis of these source-dependent effects. The sulfation codes, chain lengths, and CS/HS ratios of dE versus s-dE perlecan remain to be characterized and represent an important direction for future work. A notable paradox was the absence of corresponding *BGLAP* upregulation in s-dE(H) despite maximal mineral deposition, likely reflecting a temporal offset—FGF-2 can drive mineralization without obligatory *BGLAP* transcription (An et al., 2015); sequential time-course sampling in future studies will clarify the precise kinetics of *BGLAP* expression relative to nodule formation.

Several limitations should be noted. First, the structural differences in perlecan HS chains—sulfation patterns, chain lengths, and potential co-occurrence of CS chains—between dE and s-dE remain uncharacterized. To address this, future studies will incorporate quantitative Blyscan assays to determine total sulfated GAG content across all experimental groups, thereby complementing the current qualitative histological observations. Furthermore, more detailed glycomic approaches, such as disaccharide composition analysis, will be explored to directly resolve the fine structural differences in HS between dE and s-dE. Second, while FGF signaling is the most parsimonious explanation, other HS-binding factors (e.g., Wnt ligands, BMPs, VEGF) (Hayes et al., 2022b) may also contribute to the differentiation phenotypes, and targeted pharmacological or genetic inhibition experiments will be necessary to indicate causality. Third, beyond biochemical signaling, enzymatic removal of HS chains may also alter the biomechanical properties of the dECM, including stiffness and elasticity. Indeed, dECM from distinct cellular sources exhibits different mechanical stiffness, which may differentially modulate the multilineage differentiation potential of expanded cells (Li et al., 2020). Future comparative analyses of dE versus dE(H), and s-dE versus s-dE(H), with respect to their mechanical properties would help determine whether the observed differentiation outcomes are partly attributable to matrix biomechanics rather than, or in addition to, altered growth factor signaling. Fourth, the differentiation-enhancing effects of s-dE(H) preconditioning have been demonstrated only *in vitro*; validation in large-animal models of osteochondral defects will be required before therapeutic application can be considered.

## 5 Conclusion

HPA selectively ablated HS chains from matrix-resident perlecan in cell-derived dECM without disrupting the core protein. The functional consequences were strikingly source-dependent: preconditioning on dE(H) attenuated stemness gene expression, impaired chondrogenic capacity, and enhanced adipogenic differentiation, with no effect on osteogenesis; preconditioning on s-dE(H) similarly enhanced adipogenesis but additionally conferred markedly superior chondrogenic and osteogenic capacities—including exclusive matrix mineralization. These opposing outcomes point to a possible mechanism whereby structural and functional differences in the HS chains of perlecan from normal versus SV40LT-immortalized IPFSCs may differentially modulate FGF and BMP signaling upon enzymatic removal. However, this mechanistic interpretation remains speculative in the absence of direct biochemical evidence characterizing HS chain composition, sulfation patterns, or ligand-binding affinity in the two matrix systems. Collectively, these findings indicate perlecan-anchored HS chains as context-dependent regulators of stem cell fate, and suggest that strategic manipulation of matrix HS architecture—guided by the cellular origin of the deposited matrix—offers a tractable approach for optimizing dECM-based platforms in regenerative medicine.

## Data Availability

The datasets used and/or analysed during the current study are available from the corresponding authors on reasonable request.

## Supplementary material

Supplementary Figure 1 Flow histogram data of MSC surface markers (CD73, CD90, CD105, CD146, and SSEA4) in expanded IPFSCs.
