# Supplementary material for: Source-dependent perlecan heparan sulfate chains in dECM modulate stem cell fate and multilineage differentiation
Source: Front Bioeng Biotechnol. 2026 Oct 21;14:1930107. doi: 10.3389/fbioe.2026.1930107 (PMC13731377; doi:10.3389/fbioe.2026.1930107)
Supplement: Supplementary file 1 [file DataSheet1.docx]

Supplementary material

Supplementary Figure 1. Flow histogram data of MSC surface markers (CD73, CD90, CD105, CD146, and SSEA4) in expanded IPFSCs.
